# Clinical Outcomes of MOK Pharmacopuncture in an Elderly Male Patient with Hypothyroidism—A Case Report and Literature Review

**DOI:** 10.3390/jpm14040331

**Published:** 2024-03-22

**Authors:** Jin-Ho Jeong, Ji Hye Hwang

**Affiliations:** 1 Namsangcheon Korean Medicine Clinic, Seoul 06656, Republic of Korea; ipomoea@empas.com; 2 Department of Acupuncture and Moxibustion Medicine, College of Korean Medicine, Gachon University, Seongnam 13120, Republic of Korea

**Keywords:** hypothyroidism, L-thyroxin, MOK pharmacopuncture, acupuncture, autoimmune thyroid disease, case report

## Abstract

Hypothyroidism is more common in women and individuals between 30 and 50 years old. This case report depicts the clinical outcomes of MOK pharmacopuncture, a type of Korean medicine treatment, for an elderly male patient with hypothyroidism who was on long-term L-thyroxine (LT4) therapy but still felt chronically lethargic and tired and was generally in poor health. A 72-year-old Korean man has been on LT4 since being diagnosed with hypothyroidism 16 years ago and has tried to discontinue hormone supplements in the past. The patient was treated with MOK pharmacopuncture, mainly at the ST10 acupoint, twice a week for four months. Following the treatment, the T3, free-T4, and TPO Ab levels and thyroiditis status on ultrasound showed improvement. Additionally, there were a normalization of ESR levels, an enhancement in the quality of life, a reduction in depression scores, an improvement in the antioxidant status, and an alleviation of major symptoms when compared to pre-treatment conditions. This case report demonstrates the potential of MOK pharmacopuncture as a complementary treatment for an elderly man with hypothyroidism who had a poor quality of life due to fatigue and lethargy despite LT4 treatment.

## 1. Introduction

Thyroid hormones are essential for adequate brain development in infants and metabolic activity regulation in adults; further, they have a wide array of effects on every organ system in the body. Hypothyroidism, a typical endocrine disorder of the thyroid gland, is caused by insufficient thyroid hormones, such as thyroxine (T4) and triiodothyronine (T3). The common symptoms of hypothyroidism include tiredness, aches, dry skin, dry hair, weight gain, constipation, cold intolerance, and a slower metabolism [1,2,3]. The most common cause of hypothyroidism is the autoimmune disorder Hashimoto’s thyroiditis (HT), also known as chronic autoimmune thyroiditis and autoimmune hypothyroidism. In treating hypothyroidism, L-thyroxine (also known as LT4 and levothyroxine) is typically prescribed first, and thyroid replacement hormones are usually well-tolerated [4]. However, this type of hormone replacement therapy has not only the inconvenience of lifelong medication but also problems, such as symptoms not improving despite normal hormone levels after taking the medication [5]. There are also reports that the excessive use of LT4 increases the risk of ischemic heart disease, such as unstable angina or acute myocardial infarction, and osteoporosis [5,6]. Therefore, there is a need to develop effective treatments with fewer side effects that can complement or replace these treatments.

A previous review on acupuncture in the treatment of thyroid dysfunction reported that acupuncture is as safe and economical as integrative therapy or alternative medicine; it also reported that acupuncture could alleviate symptoms, improve relevant biomarkers, and be an alternative and complementary treatment method to help patients to cope with thyroid dysfunction [7]. Pharmacopuncture is a new form of acupuncture that combines acupuncture with the injection of herbal medicines. Compared with traditional acupuncture, the main advantages of pharmacopuncture are more rapid effects, the ease in the dosage adjustment, and additional synergistic effects from acupuncture with the injection of herbal medicine extracts [8,9]. In addition, because they are absorbed directly without passing through the gastrointestinal tract, herbal medicines can be used for patients who have difficulty in swallowing or who refuse to take them orally. Since it was first introduced to KM by Nam Sang-cheon in 1967, pharmacopuncture has become one of the main treatment methods for KM and is widely used for various diseases [8,9].

MOK is the name of a pharmacopuncture medicine modified from OK (BUM, *Bovis Calculus–Fel Ursi–Moschus*), which has anti-inflammatory and immunoregulatory effects [9]. Among the pharmacopuncture medicines, MOK is used in Korean medicine (KM) clinical practice to treat heart and thyroid diseases and stress by controlling the fire meridians. MOK, a pharmacopuncture drug consisting of 10 herbs, has been reported to have anti-inflammatory, antioxidant, and immunoregulatory effects [10,11,12,13]. MOK pharmacopuncture treatment was first mentioned as a pharmacopuncture treatment for thyroid disease. To date, there are few pharmacopuncture solutions, other than MOK, that are consistently used for thyroid disease in KM clinical practice. The MOK pharmacopuncture treatment method for thyroid diseases is described in detail in the literature on clinical pharmacopuncture treatment, and clinical treatment cases for patients with various thyroid diseases, such as hyperthyroidism and goiter, can be found in [14]. However, published clinical research on MOK pharmacopuncture for thyroid dysfunction is lacking. Therefore, to confirm the efficacy of MOK pharmacopuncture for clinical use in thyroid diseases and to accumulate scientific evidence, we report the clinical results of an elderly male patient with hypothyroidism who was in poor condition despite thyroid hormone treatment and who received MOK pharmacopuncture for four months concurrently with the existing treatment.

## 2. Case Report

We report the case of an elderly male patient (aged 72 years; 166.2 cm; 66.4 kg) who was diagnosed with hypothyroidism 16 years before presenting to our clinic and had been taking LT4 since his diagnosis. He felt excessive lethargy and fatigue despite taking this medication. At the time of his visit to us, he was taking 5 μg of oral Synthyroxine daily on an empty stomach.

His previous medical history included depression, which was diagnosed in 2004; a herniated lumbar disc, diagnosed in 2010; and inflammation in the middle ear, diagnosed in 2010. In February 2023, he was diagnosed with high blood pressure. He is currently taking a thyroid hormone analog, depression medication, and high-blood-pressure medication every day. He sometimes takes headache medication for headaches. His family history is unremarkable. He experienced the drug-related side effects of insomnia after taking Actifed (pseudoephedrine/triprolidine) tablets. He reported walking for 1 h every day and sometimes hiking. Additionally, he typically drinks 1 cup of coffee a day and 2 cups of soju at a time about once a week; he is a nonsmoker.

Before starting the treatment at our clinic, he underwent a physical and ultrasonographic examination of his neck. In December 2018, the patient underwent a thyroid ultrasound at the family medicine department of a local hospital. The ultrasound revealed coarse, heterogeneous parenchymal echogenicity in both lobes, as well as multiple cysts of varying sizes (2 on the right and 2 on the left). The cysts were well-demarcated, round, and less than 10 mm in size and were classified as Korean Thyroid Imaging Reporting Data System (K-TIRADS) 2 (benign). Additionally, there were increased vascularity and no lymph-node abnormalities in both sides of the neck. An ultrasound performed prior to the start of our treatment showed no change from the 2018 findings and diagnosis of bilateral diffuse parenchymal thyroid changes and diffuse thyroiditis. He had high levels of the thyroglobulin antibody (TG Ab) and thyroid peroxidase antibody (TPO Ab), suggesting Hashimoto’s thyroiditis as the cause of his hypothyroidism.

He tried to discontinue hormone supplements in the past, but he could not stop taking these drugs because of the consequent increased fatigue and lethargy; he had recently experienced excess fatigue and was in suboptimal physical condition despite taking medications; therefore, he wanted to undergo KM treatment, primarily as an alternative for taking thyroid hormones. Given the patient’s age and medical history, we decided to prioritize a combination of LT4 and KM treatment rather than discontinuing the LT4. The patient was taking a lot of western medications and had experienced drug-related side effects, so he preferred to use other KM treatment methods rather than taking herbal medicines. He provided informed consent and received MOK pharmacopuncture treatment while continuing to take his existing LT4 medication.

This case report was generated based on a retrospective chart review subject to the patient’s agreement to publication. The Institutional Review Board of Gil Korean Medical Hospital, affiliated with Gachon University (IRB No. GIRB-24-102), sanctioned this research.

### 2.1. Intervention

The patient was treated with MOK pharmacopuncture twice a week for 4 months concurrently with the existing LT4 treatment. The MOK extract (53.1 mg/mL in a sealed vial) was manufactured at the Namsangcheon Herbal Medicine Dispensary (Yongin, Republic of Korea), an extramural facility meeting Korean Good Manufacturing Practice standards, and comprised 10 substances as follows: *Hominis Placenta* (2 mg/mL), *Moschus* (0.5 mg/mL), *Fel Ursi* (0.3 mg/mL), *Bovis Calculus* (0.3 mg/mL), *Cortex Phellodendri* (10 mg/mL), *Scutellariae Radix* (10 mg/mL), *Sophorae subprostratae radix* (10 mg/mL), *Pulsatilla koreana* (10 mg/mL), *Aucklandiae Radix* (5 mg/mL), and *Aquilaria agallocha* (5 mg/mL).

**Figure 1:**
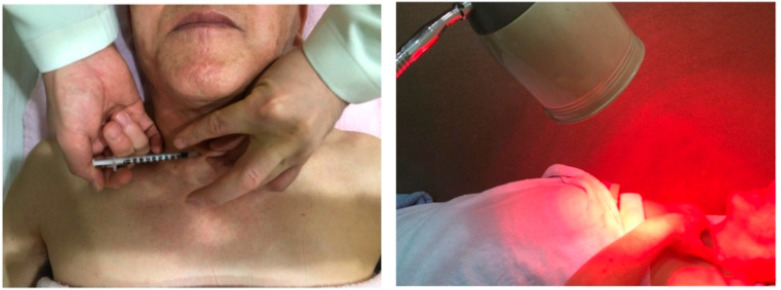
The procedure of MOK pharmacopuncture at ST10.

During the procedure, 0.2 mL of MOK was injected on either the left or right side of the *Shuitu* (ST10), which is near the thyroid gland. The left and right sides were alternated at each visit. Infrared irradiation was administered using a lamp for 10 min while manually pressing the procedure area (Figure 1).

### 2.2. Examination

The patient underwent an examination before and after the MOK pharmacopuncture. First, we performed thyroid function tests using blood samples; this included assessments of basic laboratory characteristics, oxidative stress (OS) markers, T3, free T4, thyroid-stimulating hormone (TSH), and thyroid autoantibodies, including thyroglobulin antibody (TG Ab), thyroid peroxidase antibody (TPO Ab), and TSH-binding inhibitor immunoglobulin (TR Ab). In the analysis of the OS markers, the serum total antioxidant status (TAS) and total oxidant status (TOS) were colorimetrically measured with kits (Rel Assay Diagnostics kit; Mega Tip, Gaziantep, Turkey) developed by Erel, and OS index (OSI) values were calculated.

Second, the quality of life (QOL), depression, primary symptoms, and body composition were analyzed. In general, the importance of the QOL and mental health in hypothyroid patients is significant. We administered the WHOQOL–BREF questionnaire and evaluated the patient’s QOL; this questionnaire comprises 26 questions spanning the four fields of physical health, mental health, social relations, and environmental health. In addition, it has two miscellaneous questions surveying the general health condition and QOL level. This patient’s condition was also assessed using the Hamilton Depression Scale (HAM-D). The 17-item HAM-D is the most widely used clinician-administered depression rating scale. The severity of the main symptoms, such as fatigue, dyspepsia, and cold intolerance, was gauged using a visual analog scale (VAS), while his anthropometric measurements and body composition were analyzed using a body mass analyzer (InBody 510, Biospace Inc., Seoul, Republic of Korea). Dinamika, based on the theory of heart rate variability (HRV), is a diagnostic device that can be used to evaluate various functional diseases and the function and condition of the human body [15,16].

This study evaluated the patient’s endocrine function and body energy using NEO Dinamika (MR Co., Ltd., Ansan, Republic of Korea).

In addition, the treatment satisfaction and response to the pharmacopuncture were evaluated. At the end of each treatment cycle, the treatment satisfaction was assessed on a 5-point Likert scale, and the adverse reactions associated with the pharmacopuncture procedure, such as pain, itching, induration, redness, and bruising occurring during the treatment period, were examined.

### 2.3. Changes in Thyroid Profiles, Thyroid Ultrasound Findings, Oxidative Stress, and Basic Laboratory Characteristics

**Figure 2:**
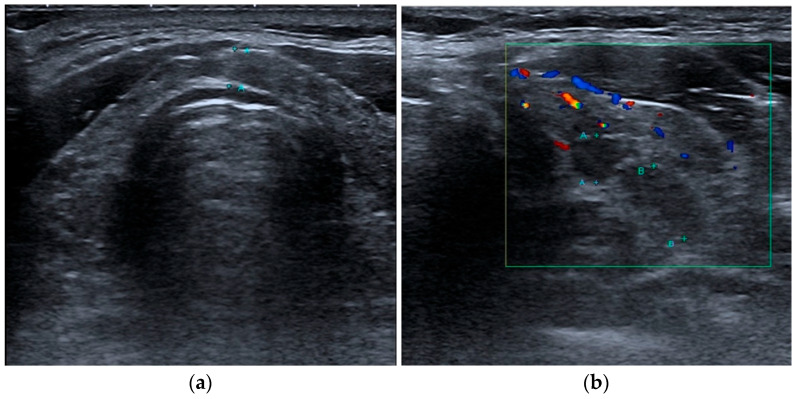
Thyroid ultrasound examination results after MOK pharmacopuncture treatment: The isthmus thickness was slightly reduced (**a**), the size of the pre-existing cysts in the left thyroid decreased, and slight parenchymal changes were observed in both thyroids, indicating an improvement in thyroiditis (**b**). A: 4.4mm distance, B: 7.5mm distance.

In the thyroid function test, T3 was lower than the normal range both before and after the treatment; free T4 and TSH were within the normal range, and after the treatment, T3 improved closer to the normal range. The level changes were as follows: Before the treatment, T3, free T4, and TSH were 65 ng/dL, 1.22 ng/dL, and 2.62 μIU/mL, respectively. After the first treatment session, T3, free T4, and TSH were 74 ng/dL, 1.32 ng/dL, and 3.72 μIU/mL, respectively. Regarding thyroid autoantibodies, the patient had significantly higher TG Ab and TPO Ab levels before the treatment. After the treatment, his TPO Ab levels reduced from 238 IU/mL to 216 IU/mL (normal range, 0–34 IU/mL), whereas his TG Ab levels marginally increased from 1247 IU/mL to 1439 IU/mL (normal range, 0–115 IU/mL). TSH-binding inhibitor immunoglobulin (TR Ab) levels were in the normal range before and after the treatment (normal range, 0–1.75 IU/L) (Table 1). In a post-treatment thyroid ultrasound of a patient with diffuse thyroiditis and multiple cysts in both sides of the thyroid gland, the size of the previously known cysts in the left side of the thyroid gland decreased compared with that of the cysts pre-treatment, the thickness of the isthmus decreased slightly, and slight parenchymal changes were observed in both sides of the thyroid gland, suggesting that the thyroiditis had improved (Figure 2).

The OS levels were reduced with the treatment, with TOS decreasing from 3.9 µmol/L to 2.3 µmol/L and OSI from 0.23 to 0.11; TAS increased from 1.7 µmol/L to 2.1 µmol/L. Among the basic laboratory characteristics, no specific abnormalities were observed in the blood and urine tests, except for a higher erythrocyte sedimentation rate (19; normal range: 2–15 mm/h), with the erythrocyte sedimentation rate improving to 5 after the treatment (Table 1).

### 2.4. Changes in QOL, Depression, VAS for the Main Symptoms, and Body Composition

The WHOQOL–BREF score improved from 57.1 before the treatment to 64.1 after the treatment. The HAM-D score improved from 20 before the treatment to 11 after the treatment. The patient’s main symptoms improved, with the VAS scores decreasing from 5 to 3 for fatigue, from 7 to 3 for dyspepsia, and from 8 to 5 for cold intolerance.

There was a slight increase in the bodyweight, and the skeletal muscle mass increased from 25.9 kg to 26.7 kg. The fat-free mass increased from 47.3 kg to 48.4 kg; the basal metabolic rate increased from 1392 kcal to 1416 kcal, and the body fat mass slightly increased from 19.1 kg to 19.2 kg, which seemed to improve the body composition (Table 1).

**Figure 3:**
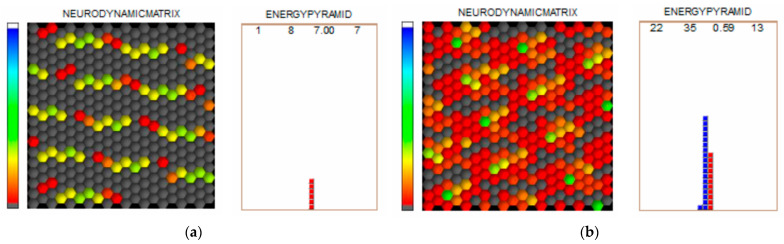
Results of the endocrine system and energy evaluations before (**a**) and after (**b**) treatment with Dinamika.

**Figure 4:**
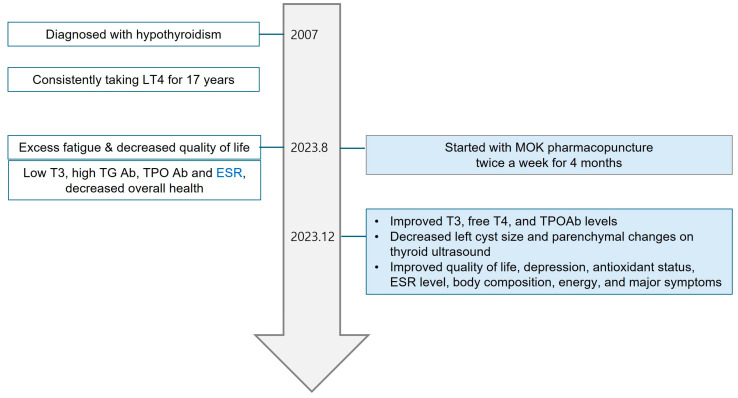
Timeline of the patient.

The endocrine system’s (hormone secretion) evaluation based on the central nervous system’s rhythm and the energy resource evaluation using Dinamika showed some improvement after the treatment compared to before the treatment (Figure 3 and Figure 4, respectively).

### 2.5. Results of Treatment Satisfaction and Response to MOK Pharmacopuncture

The treatment satisfaction score after the treatment was 4 on a 5-point Likert scale. During the treatment, procedural pain occurred about 5 times at the beginning of the therapy, itching occurred 2 times at the beginning of the treatment, and induration, redness, and bruising occurred 3 times at the beginning. Still, all were mild, and the symptoms improved without any treatment.

## 3. Discussion

Primary hypothyroidism is up to eight to nine times more common in women than in men, with a peak incidence observed between the ages of 30 and 50 years [17]. A study reported that men with hypothyroidism are generally physically weaker than women with hypothyroidism [18]. Generally, the importance of QOL and mental health in hypothyroid patients is considerable [19]. We reported the treatment responses, including changes in QOL and depression, to MOK pharmacopuncture for an elderly male patient with hypothyroidism.

In both KM and traditional Chinese Medicine (TCM), it is believed that thyroid disease is caused by an imbalance in the body’s yin and yang and that it is possible to improve the symptoms while treating the cause by normalizing the imbalance in the immune function that causes it [13]. Because the patient was elderly and taking multiple Western medications, we chose pharmacopuncture therapy, which is simple and does not require taking herbal medicines. We used pharmacopuncture, a new form of acupuncture, using MOK extract, mainly at the ST 10 point, which is near the thyroid gland. Currently, pharmacopuncture is widely used, in KM for various diseases, as one of the main therapeutic modalities [20]. MOK is a widely used pharmacopuncture medicine for treating thyroid diseases. It has anti-inflammatory, antioxidant, and immunomodulatory effects, as confirmed by cell experiments on mouse splenocytes [12] and macrophages [13]. Previous studies have also shown its in vivo anti-hypothyroid [11] and anti-hyperthyroid [10] effects. In KM and TCM, ST10 is associated with treatments for thyroid disorders, pharyngitis, shortness of breath, asthma, tracheitis, and whooping cough [9]. MOK pharmacopuncture therapy can stimulate ST10, which has a therapeutic effect on thyroid diseases. Additionally, the drug can be directly applied to the affected area, improving the treatment’s efficiency. In a previous study on hypothyroidism-induced rats, it was suggested that MOK pharmacopuncture treatment could improve the pathological progression of hypothyroidism through multiple actions, including the normalization of the hypothyroidism-induced thyroid hormone imbalance, stimulation of the antioxidant defense system, and regulation of the T helper (Th)1/Th2 imbalance [11]. Therefore, we examined not only thyroid profiles but also the antioxidant capacity and inflammation.

Since the 1970s, LT4 has become the standard thyroid hormone replacement therapy in patients unable to produce their thyroid hormones because of autoimmune, congenital, or iatrogenic causes [21,22]. The thyroid gland produces the thyroid T3 and T4 hormones. Both are used to treat hypothyroidism, but T4 is preferred because T3 is rapidly absorbed via the intestine [22]. Although LT4 has become one of the most widely used drugs worldwide [21], it remains controversial whether it is the best way to replace thyroid hormones therapeutically. Small groups of people on LT4 monotherapy have reported not feeling as if they had achieved their premorbid well-being [23].

Our patient had been on LT4 therapy for a long time but felt unhealthy and in poor condition; he had attempted to stop taking LT4 several times, and his blood tests showed lower T3 levels and significantly higher levels of thyroid autoantibodies TG Ab and TPO Ab. In our study, the patient’s T3 level, which was lower than normal, improved to closer to normal after the treatment, and the free-T4 and TSH levels slightly increased within the normal range. These results are not entirely consistent with our previous in vivo study on hypothyroidism, which showed that MOK pharmacopuncture regulated TFT through TSH reduction and T4 and T3 increases [11]. However, it has been demonstrated that MOK pharmacopuncture regulates TFT by increasing T4 and T3.

Autoantibodies against TPO, Tg, and TSHR characterize autoimmune thyroid disease. The prevalence of anti-TPO and anti-Tg antibodies is high in patients with Graves’ disease (GD) and HT; however, anti-TSHR antibodies are common in GD patients but relatively rare in HT patients [24]. Our patient showed high serum TG Ab and TPO Ab levels, and after the MOK treatment, he had reduced TPO Ab levels and slightly increased TG Ab levels. Tg Ab, like TPO Ab, is an autoantibody commonly found in autoimmune thyroid diseases; however, it is diagnostically less valuable than TPO Ab. Patients with persistently positive TPO Abs are reportedly more likely to develop recurrent or persistent hyperthyroidism [25,26]. Therefore, our improvement in TPO Ab levels after MOK pharmacopuncture is notable and can be thought to demonstrate the potential of MOK pharmacopuncture as a treatment for autoimmune thyroid disease. The mechanism of this result can be linked to the immunomodulatory effects of MOK pharmacopuncture, which have been confirmed in our previous in vitro [12] and in vivo studies [11].

OS is involved in the pathogenesis and complications of many diseases and may also be related to hormonal derangement. Because both hyperthyroidism and hypothyroidism are associated with OS in animals and humans, thyroid hormones play particularly significant roles, considering their various hormonal influences on the antioxidant balance [27]. A previous study on the associations of HT with thyroid autoantibodies and OS reported that OS increased continuously during the developments of subclinical hypothyroidism and overt hypothyroidism in patients with HT [28]. Our results showed that MOK pharmacopuncture could alleviate OS with decreased TOS and OSI and increased TAS. This result can be linked to the antioxidant effects of MOK pharmacopuncture, which have been confirmed in our previous in vitro [12,13] and in vivo studies [10,11].

Hypothyroidism decreases the metabolic rate and impairs the release of enzymes, leading the body to store more calories than it expends [26]. Our patient’s metabolic rate improved and so did his body composition, particularly the muscle-to-fat ratio, with increased muscle mass and decreased body fat. In addition, after the MOK herbal acupuncture treatment, improvement was confirmed in the inflammation level (ESR) and thyroiditis ultrasound findings, whereas endocrine function and energy improvements were confirmed through Dinamika diagnosis.

In general, the importance of the QOL and mental health in patients with hypothyroidism is significant [19]. The effect of autoimmunity thyrotoxicosis on the quality of life of patients with autoimmune hypothyroidism has been determined [29]. Another study that examined the relationship between QOL and hypothyroidism found that QOL, in most patients with hypothyroidism, was at an intermediate level [30]. Patients with hypothyroidism commonly experience depression as a comorbidity and, in most cases, develop treatment-resistant depression. It has been reported that levothyroxine improves the action of antidepressants and strengthens the effect for treating and improving depression symptoms [31]. However, the patient in this case suffered from a decreased quality of life and depression despite LT4 treatment. It was confirmed that the patient’s quality of life improved and his depression was alleviated after the concurrent treatment with MOK pharmacopuncture.

The thyroid gland is a thermostat for the body because it helps to regulate heat. People with hypothyroidism tend to have low body temperatures and low tolerance for cold. As thyroid hormones are involved in metabolism and digestion, a problem with the thyroid can result in gastrointestinal symptoms. Fatigue is also a common symptom of hypothyroidism [4,32]. Treatment for hypothyroidism usually improves energy levels and overall body functioning. In our patient, the MOK pharmacopuncture treatment resulted in improvements in the main symptoms of hypothyroidism, such as cold intolerance, dyspepsia, and fatigue.

After a brief procedure, the patient did not experience any severe adverse reactions during or immediately after the process, and no liver or kidney damage was noted in the blood tests. This establishes the usefulness and safety of the MOK pharmacopuncture treatment. Although our patient’s T3, free-T4, and TPO Ab levels; antioxidant and inflammation statuses; major symptoms; quality of life; and depression improved, MOK pharmacopuncture alone has limitations in normalizing hormone levels quickly without combination with other medications. There was a follow-up phone call after 1 month to determine whether all the improvements were maintained.

Hypothyroidism, caused by autoimmune thyroid diseases, such as Hashimoto’s thyroiditis, is known not to permanently restore thyroid function, and it is known that some patients in younger age groups return to normal function after a period of thyroid hormone treatment or without any special treatment [33]. The patient in this case was 72 years old and had been taking hormones for 16 years. As his overall condition had deteriorated, it was judged to be unreasonable to quickly reduce or stop the hormone dose after a short period of four months of the MOK pharmacopuncture treatment. Therefore, only the effect of the combination treatment was investigated, and this report was limited to a single case. Considering a longer treatment period and observing changes in symptoms and QOL, it may be necessary to consider reducing the existing drug dose. In the future, to reveal the optimal mode of the MOK pharmacopuncture for thyroid diseases and definite therapeutic effects and mechanisms, MOK pharmacopuncture should be conducted through long-term treatment studies, more case studies targeting multiple patients, studies on the combination treatment of herbal medicines and MOK pharmacopuncture, and clinical trials.

## 4. Conclusions

MOK pharmacopuncture demonstrates potential as a complementary or alternative treatment for hypothyroid symptoms in patients dissatisfied with LT4 treatment. It improves T3, free-T4, and TPO Ab levels; thyroiditis; and QOL. It also alleviates depression, reduces OS, and improves body composition in hypothyroidism. Further research should be performed to elucidate the optimal modality of the MOK pharmacopuncture.

## Figures and Tables

**Table 1:** Treatment results.

			Before Treatment	After Treatment	Normal Range
**Thyroid Profiles**	Thyroid Function Test	T3 (ng/dL)	65	74	80–200
		Free T4 (ng/dL)	1.22	1.32	0.92–1.68
		TSH (µIU/mL)	2.62	3.72	0.25–5
	Thyroid Autoimmune Antibody	TG Ab (IU/mL)	1247	1439	0–115
		TPO Ab (IU/mL)	238	216	0–34
		TR Ab (IU/L)	<0.80	<0.80	0–1.75
**Oxidative Stress**		TOS (µmol/L)	3.9	2.3	≤4.9
		TAS (mmol/L)	1.7	2.1	≥1.4
		OSI	0.23	0.11	≤0.35
**Laboratory Characteristics**		ESR (mm/h)	19	5	2–15
**WHOQOL–BREF Score**			57.1	64.1	
**HAM-D Score**			20	11	
**VAS Score**		fatigue	5	3	
		dyspepsia	7	3	
		cold intolerance	8	5	
**Body Composition**		BW (kg)	66.4	67.5	
		FFM (kg)	47.3	48.4	
		SMM (kg)	25.9	26.7	
		BFM (kg)	19.1	19.2	
		BFP (%)	28.7	28.5	
		BMR (kcal)	1392	1416	

TSH: thyroid-stimulating hormone; TG Ab: thyroid autoantibodies, including thyroglobulin antibody; TPO Ab: thyroid peroxidase antibody; TR Ab: TSH-binding inhibitor immunoglobulin; TOS: total oxidative status; TAS: total antioxidant status; OSI: oxidative stress index; ESR: erythrocyte sedimentation rate; WHOQOL: World Health Organization Quality of Life assessment instrument; HAM-D: Hamilton Depression Scale; VAS: visual analog scale; BFM: body fat mass; SMM: skeletal muscle mass; BFP: body fat percentage; FFM: fat-free mass; BMR: basal metabolic rate.

## Data Availability

The data presented in this study are available on reasonable request from the corresponding author.
